# Endocrine Disruption: Computational Perspectives on Human Sex Hormone-Binding Globulin and Phthalate Plasticizers

**DOI:** 10.1371/journal.pone.0151444

**Published:** 2016-03-10

**Authors:** Ishfaq A. Sheikh, Rola F. Turki, Adel M. Abuzenadah, Ghazi A. Damanhouri, Mohd A. Beg

**Affiliations:** 1 King Fahd Medical Research Center, King Abdulaziz University, Jeddah, Kingdom of Saudi Arabia; 2 KACST Technology Innovation Center in Personalized Medicine, King Abdulaziz University, Jeddah, Kingdom of Saudi Arabia; 3 Department of Obstetrics and Gynecology, Faculty of Medicine, King Abdulaziz University, Jeddah, Kingdom of Saudi Arabia; Hormel Institute, University of Minnesota, UNITED STATES

## Abstract

Phthalates are a class of high volume production chemicals used as plasticizers for household and industrial use. Several members of this chemical family have endocrine disrupting activity. Owing to ubiquitous environmental distribution and exposure of human population at all stages of life, phthalate contamination is a continuous global public health problem. Clinical and experimental studies have indicated that several phthalates are associated with adverse effects on development and function of human and animal systems especially the reproductive system and exposures during pregnancy and early childhood are by far of utmost concern. Sex hormone-binding globulin (SHBG) is a plasma carrier protein that binds androgens and estrogens and represents a potential target for phthalate endocrine disruptor function in the body. In the present study, the binding mechanism of the nine phthalates i.e. DMP, DBP, DIBP, BBP, DNHP, DEHP, DNOP, DINP, DIDP with human SHBG was delineated by molecular docking simulation. Docking complexes of the nine phthalates displayed interactions with 15–31 amino acid residues of SHBG and a commonality of 55–95% interacting residues between natural ligand of SHBG, dihydrotestosterone, and the nine phthalate compounds was observed. The binding affinity values were more negative for long chain phthalates DEHP, DNOP, DINP, and DIDP compared to short chain phthalates such as DMP and DBP. The Dock score and Glide score values were also higher for long chain phthalates compared to short chain phthalates. Hence, overlapping of interacting amino acid residues between phthalate compounds and natural ligand, dihydrotestosterone, suggested potential disrupting activity of phthalates in the endocrine homeostasis function of SHBG, with long chain phthalates expected to be more potent than the short chain phthalates.

## Introduction

Endocrine disrupting chemicals (EDCs) are man-made industrial compounds contaminating our ecosystem that have been associated with adverse effects on the endocrine homeostasis leading to abnormal developmental patterns, immunological problems, cancers, neurodevelopmental delays, and reproductive problems in the human population [1–3]. Thousands of chemicals are manufactured annually in the world and about a 1000 of them are estimated to have potential endocrine disrupting properties [4].

Phthalates are a family of environmentally pervasive and high volume production plasticizer chemicals, many of which have endocrine disrupting activity with harmful effects on different systems of the human body including the reproductive system (reviewed in [5]). Global plasticizer consumption was about 14 billion pounds in 2011 and phthalate contribution to this was 87% [6]. Because of the large scale production and wide commercial use, phthalates are ubiquitously distributed in the environment. Phthalate plasticizers are esters of phthalic acid and are short- or long-chain compounds based on their alkyl chains [7]. Both types of phthalate esters are linked non-covalently to industrial materials and readily leach into the environment.

Several phthalate esters have been the focus of evaluation for the risks to human reproduction by National Toxicology Program of the United States Environmental Protection Agency and the European Union [8–9]. These important phthalate EDCs are, dimethyl phthalate (DMP), dibutyl phthalate (DBP), di-iso-butyl phthalate (DIBP), butylbenzyl phthalate (BBP), di-*n*-hexyl phthalate (DNHP), di-2-ethylhexyl phthalate (DEHP), di-*n*-octyl phthalate (DNOP), di-iso-nonyl phthalate (DINP), and di-iso-decyl phthalate (DIDP). From these DMP and DBP are short-chain phthalates and are used in aerosols, perfumes, creams, cosmetics, nail polishes, house fragrances, baby lotions, excipients for medications etc. [10]. Long chain phthalates such as BBP, DNHP, DEHP, DNOP, DINP, DIDP are frequently used as plasticizers in polyvinyl chloride plastics, adhesives, food packaging, medical equipments and several commercial and household products such as dolls, toys, shoes, tablecloths, floor tiles, furniture upholstery, etc. Thus, phthalate contamination is a global and ubiquitous public health problem and the human population is exposed at home, office, farm, and everywhere else through food, water, air, and skin absorption on a continuous basis.

A study on 163 children of 2–36 months age revealed that all children had detectable levels of one or more phthalates, with 80% of children having mixture of seven or more phthalate compounds in their body [11]. A recent study [12] on 72 commonly used food samples from the market in Albany, New York revealed that every food sample had detectable levels of one or more phthalate compounds of the nine phthalates assayed, including the seven from above indicated phthalates. The frequencies of presence in food samples were DMP (37%), DBP (31%), DIBP (55%), BBP (54%), DNHP (15%), DEHP (74%) and DNOP (12%). This is of immense significance in view`of a report from the Centers for Disease Control, Atlanta, USA that shows about 100% of the population in the United States has detectable levels of one or more of the phthalate compounds [13].

Epidemiological and experimental studies on the adverse effects of phthalate chemicals on development and function of human and animal systems especially the reproductive system have been recently reviewed [5,14–15]. Phthalate exposure in men has been associated with cryptorchidism, hypospadias, gynecomastia, abnormal spermiogram and sperm DNA damage, and abnormal levels of prolactin, LH, FSH, testosterone, free androgen index, estradiol, and sex hormone binding globulin (reviewed in [15]). Phthalate exposure in women has been related with subfertility, endometriosis, leiomyomas, breast cancer, high rates of miscarriage, delayed or preterm gestation, and pregnancy complications such as anemia, toxemia, and preeclampsia (reviewed in [5,14,16]). Early pregnancy exposure may lead to shorter anogenital distance (AGD) in male infants [17,18] and less masculine play behaviors in boys [19] besides lower mental and physical development scores [20] and attention deficit hyperactivity disorder [21] in children. Experimental studies on phthalates in rodent models have shown a multitude of symptoms called the phthalate syndrome which in several aspects resembled the effects of phthalate exposure in human males (reviewed in [5,15,22]). The phthalate syndrome in rats is characterized by malformations in male organs (epididymis, vas deferens, seminal vesicles, prostate, external genitalia), cryptorchidism, retention of nipples/areolae, and reduced AGD [22].

The mechanisms by which EDCs, including phthalates, exert their actions are not clear and are thought to be through the nuclear hormone receptor signaling [1]. However, nonnuclear steroid receptors, orphan receptors, nonsteroid receptors, enzymatic pathways, and other mechanisms regulating the endocrine and reproductive functions have also been proposed to mediate the endocrine disrupting activity [1]. Sex hormone-binding globulin (SHBG) is a high molecular weight plasma protein that binds androgens and estrogens and plays a key role in maintaining the balance between unbound and bound sex steroids [23]. Owing to the high ligand-binding affinity, SHBG acts as a major carrier protein for steroids in the blood, and any changes in SHBG levels effects the distribution and access of these molecules to target tissues. Besides natural steroid hormones such as dihydrotestosterone, testosterone, and estradiol, SHBG has also been shown to bind several EDCs including phthalates esters [24–26]. Binding of the EDCs such as phthalate esters to SHBG in the body represents a potential way of interfering in the natural ligand-protein interactions and thus leading to harmful ramification for the normal functioning of the steroid target organs. Molecular modelling of zebra fish homolog of SHBG with several EDCs has been reported [27–29]. Recently, docking of many phthalates with androgen, progesterone, estrogen and peroxisome proliferating-activated receptors (PPARs) has also been reported [30–31]. However, molecular modelling studies of phthalate esters with human SHBG are apparently not available.

The objective of the present study was to compare the structural binding characteristics of the above mentioned nine phthalates with SHBG using computational approaches. The binding mechanism of the nine EDCs with human SHBG was delineated by molecular docking simulation, and the comparisons of the distinctive binding pattern and the interacting residues was done.

## Materials and Methods

**Fig 1:**
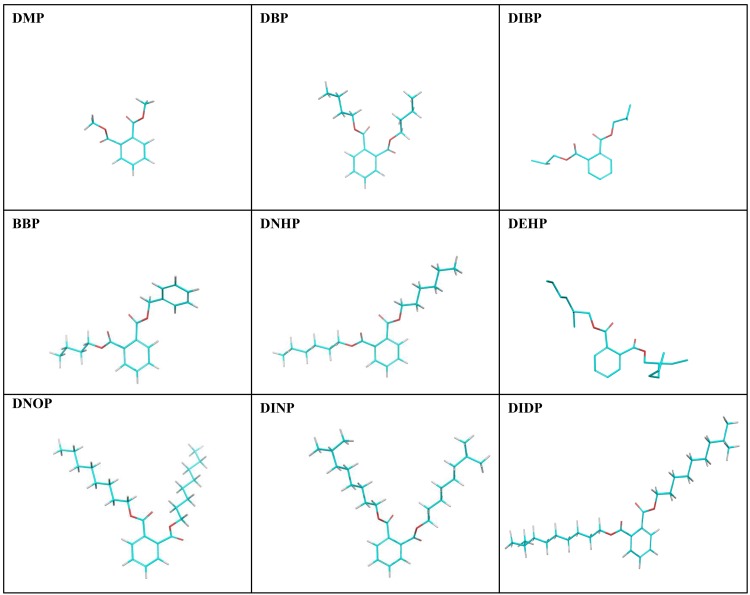
Two dimensional representation of nine phthalates i.e. dimethyl phthalate (DMP), dibutyl phthalate (DBP), di-iso-butyl phthalate (DIBP), butylbenzyl phthalate (BBP), di-*n*-hexyl phthalate (DNHP), di-2-ethylhexyl phthalate (DEHP), di-*n*-octyl phthalate (DNOP), di-iso-nonyl phthalate (DINP), and di-iso-decyl phthalate (DIDP).

Schrodinger 2015 suite containing series of modules along with Maestro 10.3 (graphical user interface) software (Schrodinger, LLC, New York, NY, 2015) was used to perform the molecular modeling of nine phthalate compounds i.e. DMP, DBP, DIBP, BBP, DNHP, DEHP, DNOP, DINP, DIDP with SHBG. The two dimensional structures of the nine phthalate compounds are illustrated (Fig 1) and the abbreviations and PubChem compound identities (CIDs) of the compounds are presented (Table 1).

**Table 1:** Nomenclature, commonly used abbreviations, and PubChem IDs of the ligands for docking study with human sex hormone-binding globulin (SHBG).

S.No	Name	Abbreviation	PubChem ID
1	Dimethyl phthalate	DMP	8554
2	Dibutyl phthalate	DBP	3026
3	Di-iso-butyl phthalate	DIBP	6782
4	Butyl benzyl phthalate	BBP	2347
5	Di-n-hexyl phthalate	DNHP	6786
6	Di-2-ethylhexyl phthalate	DEHP	8343
7	Di -n- octyl phthalate	DNOP	8346
8	Di-iso-nonyl phthalate	DINP	590836
9	Di-iso-decyl phthalate	DIDP	33599
10	Dihydrotestosterone	DHT	10635

### Protein selection and preparation

The Protein Data Bank (PDB; http://www.rcsb.org/) was searched and the three-dimensional structure of human SHBG (PDB code: 1D2S) at 1.55 Å. resolution was retrieved. The SHBG crystal structure was co-complexed with its natural ligand, dihydrotestosterone (DHT). The crystal structure was prepared by utilizing the protein preparation wizard workflow of Schrodinger after importing the structure into the docking software Glide (Schrodinger suite 2015–3; Schrodinger, LLC). Water molecules were removed and hydrogen atoms were added during the preparation protocol of the crystal structure and loops and missing side chains were built using Prime 3.0 module. Optimization of H-bond particularly for Asp, Glu, and His hydroxyl containing residues was then done. This was followed by optimization of the hydrogen bonding network and finally the OPLS_2005 force field was used for a geometry optimization to a maximum root-mean-square deviation (rmsd) of 0.30 Å. The bound ligand, DHT in crystal complex was selected and used for docking of the indicated nine phthalate esters and grid boxes were generated.

### Ligand preparation and conformational search

Maestro 10.3 (Maestro, version 10.3, Schrodinger, LLC) was used for drawing ligand structures as shown (Fig 1). Ligands were prepared using LigPrep module (Schrodinger 2015: LigPrep, version 3.1, Schrodinger, LLC). Correct molecular geometries and ionize at biological pH 7.4 were obtained by using the OPLS-2005 force field software. Retention of specific chirality and generation of least energy conformations was done using OPLS-2005 software.

### Induced fit docking

Schrodinger’s Induced Fit Docking (IDF) module with Prime program was utilized for docking analyses of the nine phthalate esters i.e. DMP, DBP, DIBP, BBP, DNHP, DEHP, DNOP, DINP DIDP. After preparation using LigPrep module, the ligands were submitted as starting geometries to IFD. The IFD has been shown to have the ability of sampling the minor changes in the backbone structure as well as robust conformational changes in side chains [32]. A softened-potential docking is performed in the first IFD stage where docking of the ligand occurs into an ensemble of the binding protein conformations. Subsequently, complex minimization for highest ranked pose is performed where ligand as well as the binding sites are free to move.

### Binding energy calculations

The calculation of binding affinity of ligands against the binding protein molecule was done using Prime module of Schrodinger 2015 with MMGB-SA function.

## Results

**Fig 2:**
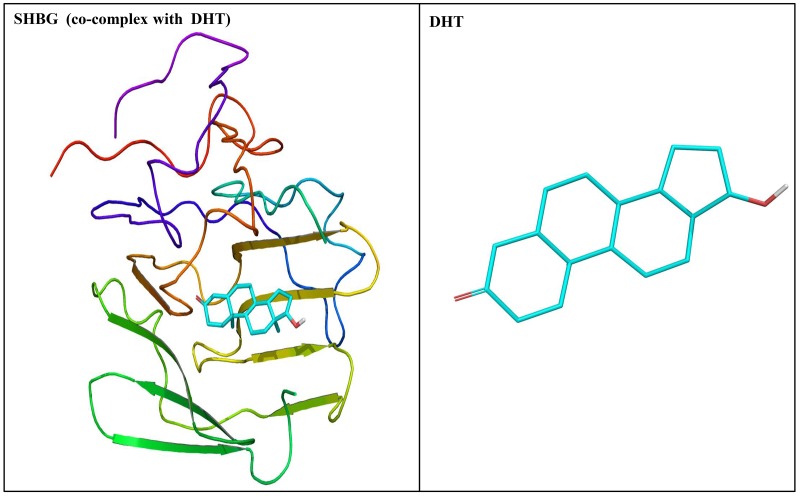
Crystal structure of human sex hormone-binding globulin (SHBG) in ribbon form representation co-complexed with dihydrotestosterone (DHT) (left panel) and two dimensional representation of DHT (right panel).

**Fig 3:**
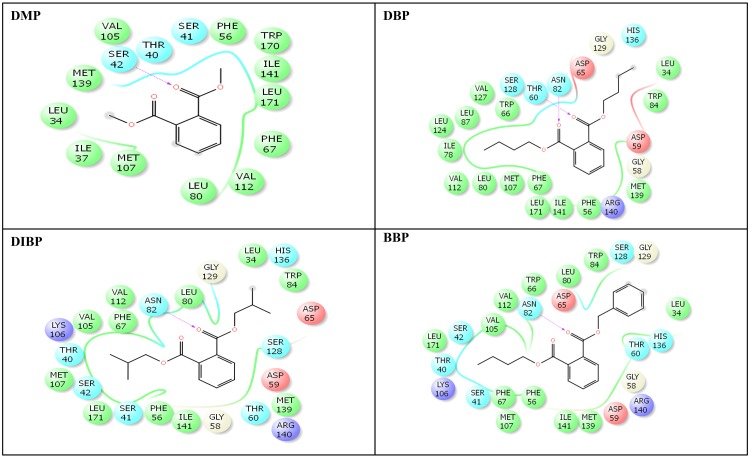
Amino-acid residues in the binding pocket of human sex hormone-binding globulin (SHBG) involved in interactions with dimethyl phthalate (DMP), dibutyl phthalate (DBP), di-iso-butyl phthalate (DIBP), butylbenzyl phthalate (BBP).

**Fig 4:**
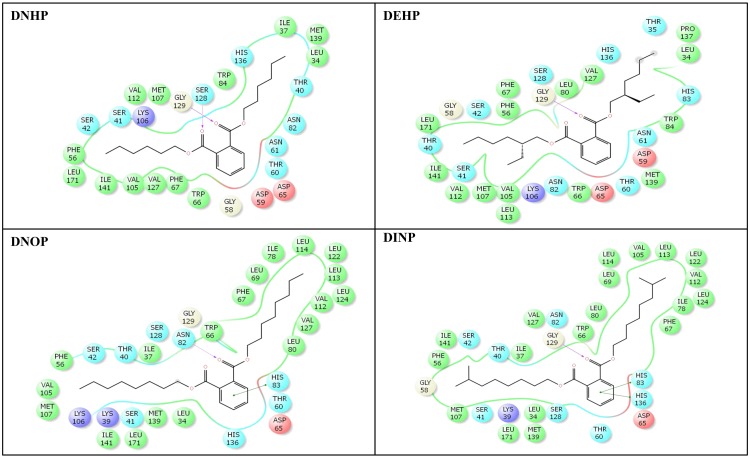
Amino-acid residues in the binding pocket of human sex hormone-binding globulin (SHBG) involved in interactions with di-*n*-hexyl phthalate (DNHP), di-2-ethylhexyl phthalate (DEHP), di-*n*-octyl phthalate (DNOP), di-iso-nonyl phthalate (DINP).

**Fig 5:**
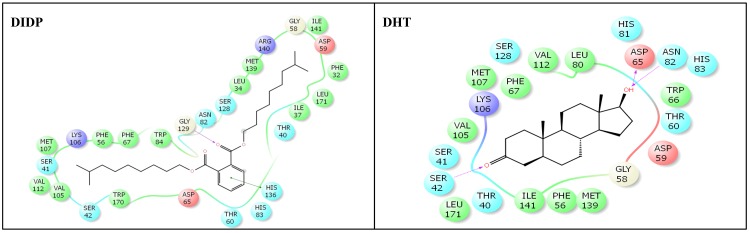
Amino-acid residues in the binding pocket of human sex hormone-binding globulin (SHBG) involved in interactions with di-iso-decyl phthalate (DIDP) and natural ligand, dihydrotestosterone (DHT).

The crystal structure of human SHBG with native ligand, DHT, is shown (Fig 2). The nine phthalate compounds i.e. DMP, DBP, DIBP, BBP, DNHP, DEHP, DNOP, DINP, DIDP chosen for docking simulation achieved successful execution of IFD against steroid binding pocket of SHBG which resulted into multiple poses for each of these compounds. The best pose for each of the phthalates was chosen for analyses. Docking complexes of the nine phthalates displayed interactions with 15–31 residues of SHBG (Figs 3–5, Table 2). For the co-complex ligand, DHT, interactions with 22 residues of SHBG were displayed by the docking complex (Fig 5; Table 2). Overall, 12–21 SHBG interacting residues were common between the natural ligand, DHT, and the nine phthalate compounds (commonality of 55–95%; Table 2). Fifteen interacting residues of SHBG (Asn-82, Asp-65, Ile-141, Leu-171, Met-107, Met-139, Phe-56, Phe-67, Ser-41, Ser-42, Ser-128, Thr-40, Thr-60, Val-105, Val-112) were common among the native ligand, DHT, and all the nine phthalate compounds (the exceptions were four residues each that were not common for DMP and DBP; Table 3). Further, there were five interacting residues of SHBG (Asp-59, Gly-58, Leu-80, Lys-106, Trp-66) which were common among DHT and the majority (6–7) of the nine phthalate compounds (not shown). One interaction displayed by residue, Leu-34, was common for all the nine phthalate compounds but not for DHT. Conversely, one interacting residue, His-81, was displayed by the DHT docking complex but not by any of the nine phthalate compounds (not shown). The Dock score, Glide score, and binding affinity values (MM-GBSA values) for the nine phthalate compounds and the bound ligand, DHT, are presented (Table 2). Among the nine phthalate compounds, DEHP, DNOP, DINP, and DIDP have the highest Dock score, and the highest Glide score which are, however, slightly lower than the native ligand, DHT. In addition, the binding affinity values (MM-GBSA values) for these four phthalates were highest among the nine phthalates and were higher than the native ligand, DHT (Table 2).

**Table 2:** Number of interacting residues, number and percentage of residues common with dihydrotestosterone (DHT), Dock score, Glide score and binding affinity values (MMGB-SA values) of nine phthalate plasticizers i.e. dimethyl phthalate (DMP), dibutyl phthalate (DBP), di-iso-butyl phthalate (DIBP), butylbenzyl phthalate (BBP), di-*n*-hexyl phthalate (DNHP), di-2-ethylhexyl phthalate (DEHP), di-*n*-octyl phthalate (DNOP), di-iso-nonyl phthalate (DINP), and di-iso-decyl phthalate (DIDP), and natural ligand dihydrotestosterone (DHT) during docking with of human sex hormone-binding globulin (SHBG).

S. No.	Ligand	Number of interacting residues	Number of interacting residues common with DHT (%)	Docking score (Kcal/mol)	Glide score (Kcal/mol)	MMGB-SA (Kcal/mol)
1	**DMP**	**15**	12 (55%)	-6.9	-6.9	-59.9
2	**DBP**	**24**	15 (68%)	-6.0	-6.0	-95.4
3	**DIBP**	**24**	19 (86%)	-7.6	-7.6	-94.6
4	**BBP**	**25**	20 (90%)	9.1	9.1	-93.4
5	**DNHP**	**26**	19 (86%)	-8.5	-8.5	-120.0
6	**DEHP**	**30**	21 (95%)	-9.4	-9.4	-132.8
7	**DNOP**	**31**	19 (86%)	-8.9	-8.9	-146.0
8	**DINP**	**31**	19 (86%)	-9.52	-9.52	-148.0
9	**DIDP**	**27**	19 (86%)	-10.12	-10.12	-147-4
10	**DHT**	**22**	22 (100%)	-12.0	-12.0	-129.8

**Table 3:** Common interacting residues of human sex hormone-binding globulin (SHBG) among co-complex natural ligand, dihydrotestosterone (DHT), and nine phthalates i.e. dimethyl phthalate (DMP), dibutyl phthalate (DBP), di-iso-butyl phthalate (DIBP), butylbenzyl phthalate (BBP), di-*n*-hexyl phthalate (DNHP), di-2-ethylhexyl phthalate (DEHP), di-*n*-octyl phthalate (DNOP), di-iso-nonyl phthalate (DINP), and di-iso-decyl phthalate (DIDP).

S.No.	Common interacting residues	S.No.	Common interacting residues
1	Asn-82#	9	Ser-41*
2	Asp-65#	10	Ser-42*
3	Ile-141	11	Ser-128#
4	Leu-171	12	Thr-40*
5	Met-107	13	Thr-60#
6	Met-139	14	Val-105*
7	Phe-56	15	Val-112
8	Phe-67		

Four amino-acid residues indicated by hatch mark (#) were not shared by DMP and four residues indicated by star (*) were not shared by DBP.

## Discussion

Human SHBG is a steroid binding protein in the plasma and is synthesized and secreted by the liver [23,33]. Majority of dihydrotestosterone, testosterone, and estradiol in the plasma are bound to SHBG with nanomolar affinity [23]. It is generally believed that the SHBG protects these steroids from rapid metabolic degradation and thus intervenes in their availability at the target tissues. Unbound or free sex steroids constitute a minor fraction (1–3%) and are considered bioavailable for target tissues. Human SHBG is a homodimer and each SHBG subunit has two laminin G-like domains, the N-terminal domain containing the steroid-binding pocket and binding sites for calcium and zinc, and the C-terminal domain containing residues for glycosylation [23].

The current study shows that several important residues of human SHBG were involved in molecular docking with each phthalate compound and together with high dock score and high binding affinity values assured good quality docking besides contributing to the stability of the ligand-SHBG docking complex. The consistent commonality of majority of SHBG interacting residues (55–95%) for bound ligand, DHT, with those of different phthalates is interesting and explained the binding of compounds with a common steroid scaffold. This commonality of residues is suggestive of the potential of majority of indicated phthalates for interference in the steroid binding function. The additional specific residues that were involved in interactions for each phthalate and for DHT suggested their importance in specific binding of the individual ligand to the steroid binding pocket of SHBG.

All the nine phthalates exhibited good binding affinity towards SHBG. However, there appears to be a dichotomy in the docking characteristics of phthalates on the basis of length of the side chain in the phthalate molecules. The binding affinity values were more negative (more stable binding) for long chain phthalates DEHP, DNOP, DINP, and DIDP compared to short chain phthalates such as DMP and DBP. The four long chain phthalates DEHP, DNOP, DINP, and DIDP bind more tightly to the SHBG comparable to native ligand DHT and, hence, are expected to be more potent endocrine disruptors of androgen and estrogen signaling. This assumes more significance in that the overall plasticizers demand in the global market is dependent on DEHP, DINP and DIDP segment which collectively constituted almost three-fourth of the total global demand in 2013 [34]. Previous experimental studies have shown that long chain phthalates like DEHP, BBP, DINP induced severe toxic effects such as shortened AGD, reduced testis weights, female like areolas/nipples, and reproductive malformations compared to no or least such effects by short chain phthalates like DMP in male rat pups [35–36]. Our results, therefore, are supportive of these experimental studies in that the Dock score, Glide score and binding affinity values (possibly indicating the interfering ability) were several order higher for long chain phthalates compared to short chain phthalates. However, besides SHBG pathway, these adverse effects could also occur through multiple other pathways including androgen receptors etc.

Previous reports on the docking simulations for phthalate ligands with SHBG are apparently not available. However, docking studies with nuclear receptors such as androgen receptor, progesterone receptor, estradiol receptor, and PPARs have shown interaction with aromatic ring and alkyl side chains of several phthalate compounds [30–31]. In vitro competitive binding studies of many phthalate esters with human SHBG have been previously reported [24–26,37]. Using solid phase binding assays, BBP and DBP were shown to have weak binding affinity for SHBG binding sites in native plasma from normal men and women [24]. In another study [25], using radioactive estradiol and human female plasma for binding assays, several phthalates including BBP, DBP, DNHP, DEHP, DNOP, DINP and DIDP were tested for their binding activity with SHBP. Phthalates were found to exhibit very low binding affinity compared to estradiol. Recently [26], similar competitive binding assays for 125 structurally diverse compounds including DMP, DBP, DIBP, BBP, DEHP, DINP were performed using human pregnancy plasma to measure binding activity against human SHBG. The indicated phthalates exhibited week binding activity to human SHBG several orders of magnitude lower than the native SHBG ligands, dihydrotestosterone, testosterone and estradiol. There seems to be a discrepancy between our docking study and the reported in vitro binding studies [24–26] showing lower affinity of many phthalate compounds to SHBG compared to the endogenous sex steroids. The reasons for this are not known. Nevertheless, even the lower affinities may be physiologically important when SHBG levels are high and endogenous estradiol and testosterone are low, such as, during prepubertal period [38–39] and in women during pregnancy [40] or when taking medications (use of contraceptives [41]). Under such conditions phthalate binding to greater available binding sites of SHBG may be important and might affect the metabolism and tissue bioavailability of natural steroids. Interestingly, a lower binding affinity of fetal mouse serum proteins for bisphenol A compared to octylphenol (bisphenol A and octylphenol are other high volume EDCs) during in vitro assays gave contrasting results during *in vivo* exposure of mice when bisphenol A was found to have severe adverse effects and octylphenol had no effect [42].

There is no single mechanism that may explain the phthalate effects on the reproductive system. For example, the toxic actions of DEHP that were thought to be occurring through PPAR alpha pathway in mice were still observed when PPAR alpha null mice were used suggesting the role of additional pathways [43]. Phthalates are generally thought to exert their adverse actions by inhibiting testosterone synthesis from the leydig cells, however, both agonistic (androgenic) and antagonistic (antiandrogenic) effects at androgen receptors have also been shown (reviewed in [44]).

Although not related to the binding of phthalates to SHBG, it is interesting to note that conflicting epidemiological reports of negative and positive relationship between serum or urinary phthalates levels and circulating SHBG levels in children and adults are available [44–49]. Higher levels of DMP and DBP in urine were positively correlated with serum levels of SHBG [45]. Free-testosterone and estradiol were found to decrease and SHBG found to increase with increasing urinary DEHP metabolite levels [46–47]. Likewise, higher DINP was associated with increased serum SHBG levels [48]. In addition, prenatal exposure to phthalates was associated with increased serum levels of SHBG in boys [49]. Conversely, a negative association of testosterone and SHBG with serum metabolites of DEHP and DINP was shown [44]. Thus, the associations between SHBG, testosterone, estradiol and phthalates are complex and could be driven by phthalates with different mechanisms under varying homeostatic conditions of the body such as childhood, adulthood, pregnancy, presence of mixture of EDCs etc. This is also particularly significant as SHBG in addition to playing role in maintenance of steroid homeostasis is involved in membrane based steroid signaling and can modulate biological actions of sex steroids at the target cell level [50].

The present computational study suggested that several phthalate compounds are able to potentially bind to human SHBG and are likely transported in the body to various steroid target organs. Displacement of the endogenous steroids from SHBG binding sites may disrupt the androgen-estrogen homoeostasis.

## Conclusions

The present study was done to delineate the structural binding characteristics of nine phthalates i.e. DMP, DBP, DIBP, BBP, DNHP, DEHP, DNOP, DINP, DIDP with SHBG using computational approaches. Docking complexes of the nine phthalates displayed interactions with 15–31 amino acid residues of SHBG and 55–95% interacting residues of natural ligand of SHBG, DHT, were common with those for the nine phthalate compounds. The binding affinity values were more negative for long chain phthalates DEHP, DNOP, DINP, and DIDP compared to that for the short chain phthalates such as DMP and DBP. The Dock score and Glide score values were also higher for long chain phthalates compared to that for the short chain phthalates. This study shows that all the nine phthalate esters were able to engage important interacting residues of SHBG during molecular interactions and, hence, can potentially displace or compete with the natural SHBG ligands such as dihydrotestosterone, testosterone, and estradiol for the availability of SHBG binding sites resulting in altered androgen-estrogen homeostasis. However, long chain phthalates may be potentially more active endocrine disruptors. Current study enhances our understanding of underlying molecular mechanism of potential interfering mechanisms of phthalates in steroid homeostasis of the human body.
